# Notes and News

**Published:** 1892-09-17

**Authors:** 


### Notes and News.

COLLECTED AND COMMUNICATED.

A delightfully Irish advertisement recently appeared in a paper of that nationality. It ran thus: "Wanted, a gentleman to undertake the sale of a patent medicine. The advertiser guarantees that it will be profitable to the undertaker." Such a guarantee for many a patent medicine would be very profitable to the public.

The *Chronicle* makes a suggestion that, in view of the continued inadequacy of the Metropolitan Fever Hospitals to accommodate all the scarlet fever cases which should be admitted, that the Board schools should be closed for awhile, since a considerable danger of children attending them from homes where there is fever exists.

A mutual-help brotherhood has been established in St. Luke's parish. Its members include skilled artisans and efficient labourers. To assist the members of the brotherhood in times of temporary depression, a labour bureau has been established, which includes a firewood business. It is to be hoped the undertaking will succeed, but the firewood chopping will become a drug in the market if adopted by all labour bureaux.

We hope that many of our readers have perused that admirable little sketch of hospital life, entitled, "Hors de Combat," which was published at the latter end of last year, and of which a notice appeared in our columns. We are glad to learn that the youthful authoresses, the Misses Armitage Southam, have received the encouragement of Miss Nightingale's favourable opinion on their work. "Your 'Hors de Combat,'" says Miss Nightingale in her letter, "of which I have given, and shall give, many copies, is an excellent book—is an admirable book, 'most musical, most melancholy,' most cheerful and hopeful, a true and bright ideal of hospital life, which ought to put spirit into people, and for which people must thank you as I do." We are glad to have the opportunity of quoting the sentiments of one whose opinion cannot cease to carry weight, as we may thus be assisting the excellent endeavours of the authoresses to interest the public in hospitals in general, and the Salford Hospital in particular.

The new buildings of the Royal Infirmary, Aberdeen, are now completed. Some of the wards are already furnished, and patients will be admitted at an early date. The Aberdeen City Hospital, also, is about to be extended. It is proposed to enlarge two of the pavilions, to double the present administrative block, and to erect a new block for laundry, disinfecting department, and offices. If all these proposals are carried out the cost is estimated at £12,800.

We are glad to learn that the London Young Women's Christian Association continues its useful work of holding evening educational classes. A new illustrated prospectus has been issued setting forth the advantages offered by the Association, and giving the addresses of over forty institutes, homes, and restaurants in London. At several of these institutes evening classes are held in book-keeping, shorthand, dress-cutting, French, music, ambulance, &c. Classes are also conducted in gymnastics and drill. The Association has in London alone nearly 17,000 members. A copy of the prospectus will be sent free on application to the Secretary, 16a, Old Cavendish Street, W.

At Middlesborough the Tees Sanitary Authorities seem to have alighted upon some most unsatisfactory cottages in view of an emergency, which, if report speaks true, will effect but little good, owing to defective drainage and undesirable site. The Hartlepool sanitary authorities interfered, but with no effect. Of course, the question is largely one of expense, and the difficulties and dangers which arise from the fact that our fever hospitals are rate-supported, should warn those who rashly desire all our medical charities to be in the same condition. A time like the present brings into notice the actual state of these fever institutions, and it is both alarming and surprising to read so many unsatisfactory reports in local papers throughout the United Kingdom.

At a recent meeting of the Balloon Society, Professor Garner, of Virginia, gave a lecture on "The Speech of Monkeys." The lecturer remarked that the mention of monkeys was usually the signal for laughter and fun; but during his study of the animals, extending over many years, he had found a very serious side to their lives, and among them were stoics, philosophers, and clowns. He defined speech as being that form of materialised thought which was restricted to such sounds as were designed to convey a definite idea from mind to mind. This was characterised in monkeys, whose sounds were voluntary, deliberate, and articulate. They understood and acted in accordance with the sounds, when imitated by the phonograph or other mechanical means, and not by signs, gestures, or physical influence. They could count, and had favourite colours. He argued that, as they could interpret sounds of human speech, they could ascribe meanings to their own; his long observation and many tests had convinced him of this. He was now about to start for the Gaboon district of West Africa, Du Chaillu's country. He had hitherto studied monkeys in captivity, but he held that a special observation of them in their dense and natural haunts, hitherto mostly untrodden by white man, would lead to practical and satisfactory results.

AT the recent International Physiological Congress, held at Liege, it was remarked that whilst of German savants there were very few, the Italian element was only conspicuous by its absence.

AN International Congress against the abuse of alcoholic drinks commenced at The Hague on the 8th inst. A fair number of English representatives of temperance societies took part in the proceedings.

IN our last issue mention of the efficacy of brine baths in cholera was made. Since then we have heard that salt taken internally has proved of use in India in cases of this disease.

AT Lochee, in Scotland, an outbreak of typhoid has occurred. All the infection is traceable to one dairy. The sale of milk from this dairy being prohibited, the owner has disposed of his sixteen cows by auction. It seems strange that a sale under such circumstances should be permitted.

IT has been decided to erect an infectious diseases hospital at Ormeskirk. The Board of Health intend to select a site immediately. We could wish that this promptitude might be followed in the south. The Isle of Wight sanitary authorities are still debating the erection of a similar institution, with little apparent likelihood of a favourable decision being arrived at.

MR. WALKER, chief engineer of the Dundee steamer *Gerona*, whose death was reported from cholera, gave a lamentable account of the harbour at Hamburgh. He stated that the men at work on the ships there are filled with alarm at the disease, and, taking to drink to drown their fears, are thus predisposed to an attack. Whilst the *Gerona* was at Hamburgh the river water was reported as shockingly bad.

IN common with numbers of other localities, the cholera has raised the vexed question of infectious hospitals at Birkenhead. At a recent meeting of the Town Council, Alderman Shaw pointed out that Birkenhead was quite unprepared to cope with any epidemic. Difficulties attendant on the question of site have been removed, and a fever pavilion is to be erected, which will be ultimately converted into a permanent structure. Had the cholera appeared in Birkenhead at an earlier date, it would have been necessary to remove the typhoid cases from the hospital to make room for cholera cases.

IN Lady Brassey's new Convalescent Home for Children, at Bexhill, we have another of those most popular institutions whose sphere of usefulness is undoubted. In the case of children, especially the convalescent home plays a most important part towards a complete cure. Lady Brassey's project, commenced, as it has been on a small scale, is one that affords an excellent example to others. The small beginnings which have great endings are far more satisfactory than those greater efforts which, starting on too large a scale, find not only their growth, but their very existence, endangered by the unweildly nature of the machinery set in motion. Subscribers to Lady Brassey's Home of one guinea only, are entitled to nominate a patient for a three weeks' visit during the year, and we trust that many friends will be found to join a useful work at so small an outlay.

